# Taguchi Method and Response Surface Methodology in the Treatment of Highly Contaminated Tannery Wastewater Using Commercial Potassium Ferrate

**DOI:** 10.3390/ma12223784

**Published:** 2019-11-18

**Authors:** Violetta Kozik, Krzysztof Barbusinski, Maciej Thomas, Agnieszka Sroda, Josef Jampilek, Aleksander Sochanik, Adam Smolinski, Andrzej Bak

**Affiliations:** 1 Institute of Chemistry, University of Silesia, Szkolna 9, 40-007 Katowice, Poland; asroda@us.edu.pl; 2 Institute of Water and Wastewater Engineering, Silesian University of Technology, Konarskiego 18, 44-100 Gliwice, Poland; krzysztof.barbusinski@polsl.pl; 3 Chemiqua Water&Wastewater Company, Skawinska 25/1, 31-066 Krakow, Poland; biuro@chemiqua.pl; 4 Department of Analytical Chemistry, Faculty of Natural Sciences, Comenius University, Ilkovicova 6, 842 15 Bratislava, Slovakia; 5 Center for Translational Research and Molecular Biology of Cancer, Maria Sklodowska-Curie Memorial Cancer Center and Institute of Oncology, Wybrzeze AK 15, 44-101 Gliwice, Poland; aleksander.sochanik@io.gliwice.pl; 6 Department of Energy Saving and Air Protection, Central Mining Institute, Plac Gwarkow 1, 40 166 Katowice, Poland; smolin@gig.katowice.pl

**Keywords:** tannery wastewater, Taguchi method, response surface methodology, central composite design

## Abstract

The potential implementation of Envifer^®^, a commercial product containing potassium ferrate (40.1% K_2_FeO_4_), for the purification of highly contaminated tannery wastewater from leather dyeing processes was proposed. The employment of the Taguchi method for optimization of experiments allowed the discoloration (98.4%), chemical oxygen demand (77.2%), total organic carbon (75.7%), and suspended solids (96.9%) values to be lowered using 1.200 g/L K_2_FeO_4_ at pH 3 within 9 min. The application of the central composite design (CCD) and the response surface methodology (RSM) with the use of 1.400 g/L K_2_FeO_4_ at pH 4.5 diminished the discoloration, the chemical oxygen demand, the total organic carbon, and suspended solids within 9 min. The Taguchi method is suitable for the initial implementation, while the RSM is superior for the extended optimization of wastewater treatment processes.

## 1. Introduction

Due to the sustained worldwide demand for leather products, the tannery industry plays a significant role in the economy of many countries, including Ethiopia, India, Pakistan, and China [1,2,3,4]. In developing countries, tanneries do not use sufficiently effective methods to treat the wastewater generated from leather production processes [1]. Although this situation has been gradually improving, in 2004, over 90% of tanneries in Ethiopia (mainly in the south of the country), did not have any wastewater treatment systems. Furthermore, such tanneries dump wastewater directly into neighborhood water reservoirs or into the ground [1]. These practices can lead to the contamination of water reservoirs and ground areas, which negatively impacts the chemical parameters of drinkable water and, therefore, residents’ health. Even in developed countries, the treatment of tannery wastewater often poses technical problems that necessitate the use of advanced and/or innovative solutions.

Tannery wastewater, such as that generated by the textile industry, is chemically complex. Depending on the reagents used, tanning processes can be vegetable- or chrome-based [5]. Regardless of the technology applied, tanning involves many intermediate steps requiring the use of large amounts of water and chemicals. The amount and quality of the produced wastewater and sludge can vary significantly [5] depending on the raw material’s quality, amount of water required, and degree of process automation or instrumentation available. The tanning process transforms skin or hide into lasting, commercially available products. It requires the use of chemicals such as acids, bases, tannins, chromium(III) salts, surfactants, syntans (phenolic, naphthalene, formaldehyde, and melamine), dyes, and other compounds [5,6,7,8,9]. All these chemicals are present in the wastewater after rinsing to a greater or lesser degree, necessitating the use of effective wastewater treatment methods. Reference data estimate that leather tanning generates 10–80 m^3^ wastewater per ton of raw material [10,11]. Usually, tannery wastewater is neutral or basic (pH 7–10.7), contains high levels of total dissolved solids (TDS, 6810–19,700 mg/L), total solids (TS, 10,265–19,775 mg/L), and suspended solids (SS, 915–5300 mg/L), and is characterized by high chemical oxidation demand (COD, 2155 to 11,154 mg O_2_/L) and biochemical oxygen demand (BOD, 630 to 2906 mg O_2_/L). The values of these parameters demonstrate its significant organic compound content. Tannery wastewater also contains sizeable amounts of ammonia nitrogen (33–335 mg/L), total chromium (11.2–95 mg/L), and sulfides (36–508 mg/L) [12]. Depending on the chemicals used and the technological processing conditions, these values may vary significantly. One can assume, however, that tannery wastewater usually has an alkaline pH, is brown in color, and contains high levels of organic matter [13]. Moreover, it contains toxic compounds such as endocrine disrupting chemicals (e.g., di-butyl phthalate, benzyl butyl phthalate, and nonylphenol) or even carcinogens (e.g., anthracene, azo dyes, hexavalent chromium, and formaldehyde) [14], which can impair microorganisms in activated sludge at the biological clean-up stage. The toxicity (genotoxicity, cytotoxicity, and mutagenicity) of tannery wastewater against *Allium cepa*, *Aliivibrio fischeri*, and *Vicia faba* has been attributed to the heavy metals (Cr, Ni) and other toxic substances present in the wastewater [15,16,17]. The tannery wastewater has aneugenic and clastogenic potential in adult male bullfrogs (*L. castesbeianus*) [18]. The current research has also confirmed a phytotoxicity, cytotoxicity, and genotoxicity of organic and inorganic pollutants contained in tannery wastewater by using *Vigna radiate L.* and *Allium cepa L*. The toxicological studies showed that tannery wastewater (containing, among others, benzoic acid, benzeneacetamide, resorcinol, phthalates) can inhibit seed germination and root growth, can cause chromosomal aberrations (stickiness, chromosome loss, C-mitosis and vagrant chromosome) and nuclear abnormalities such as micronucleated and binucleated cells [19].

When raw tannery wastewater is discharged into water reservoirs, its brown color reduces the penetration of sunlight into water, thus impairing photosynthesis and reducing the dissolved oxygen content, which negatively impacts aquatic organisms [20]. In addition, tannery wastewater significantly affects the eutrophication of water reservoirs [21] and leads to the deposition of heavy metals in bottom sediments. In particular, untreated wastewater can have chrome and iron concentrations of 110–178 mg/kg and 4800–7250 mg/kg, respectively, compared to 5.6 mg/L and 3.2 mg/L for treated wastewater discharged into water reservoirs [22]. Tannery wastewater also increases water salinity [23], inhibits nitrification [5], and affects froth formation on the water surface due to the presence of vegetable dyes, saponins, and protein fractions that do not undergo biodegradation during treatment [24]. Consequently, tannery wastewater must be subjected to effective treatment methods before it can be safely discharged into water reservoirs. 

The first stage of treatment usually uses physical-chemical processes, which are then followed by biological methods. For the first stage, coagulation, flocculation and sedimentation [25], Fenton and photo-Fenton reagent-based processes [26,27,28,29], wet air oxidation [30], ultrafiltration, and reverse osmosis [31,32,33] processes can be used, among others. Biological processes employ the activated sludge and the up-flow anaerobic sludge blanket processes [34]; however, chemical compounds might impede biochemical oxidation processes at the initial clean-up stage. 

Following the core principles of ‘green chemistry’, increased attention is being paid in both research and industrial practice to the use of highly effective reagents that do not harm the natural environment. The use of potassium ferrate (K_2_FeO_4_) fits well with this strategy. K_2_FeO_4_ exhibits a double mechanism of action consisting of the oxidation and the coagulation of impurities present in processed wastewater. The oxidizing agent is Fe^6+^, which is reduced to Fe^3+^ during the oxidation of organic substances (as well as some inorganic substances). The redox potentials of Fe^6+^ in acidic and alkaline milieus are 2.2 V and 0.7 V, respectively [35]. In an aqueous environment, Fe^3+^ precipitates as hydrated Fe(OH)_3_, which, due to its high surface area, may adsorb impurities present in the wastewater. 

Potassium ferrate has been used to treat both textile and communal wastewater. For wastewater samples collected from a carpet factory (COD = 1600 mg O_2_/L, turbidity = 554 NTU, TSS (Total Dissolved Solids) = 280 mg/L) K_2_FeO_4_ treatment achieved COD, turbidity, and TSS removal efficiencies of 86, and 89%, respectively, under the optimum conditions (for COD: K_2_FeO_4_ = 160 mg/L, pH = 4, for turbidity: K_2_FeO_4_ = 165 mg/L, pH = 4 and for TSS: K_2_FeO_4_ = 150 mg/L, pH = 4.5) [36]. In synthetic textile wastewater containing Acid Green 16 (AG 16 = 20 mg /L, color = 66 mg Pt/L, DOC = 394 mg/L) the application of potassium ferrate under the optimum conditions (pH = 2, time = 50 min, K_2_FeO_4_ = 125 mg/L) achieved 98, 88, and 37% removal of AG16, color, and DOC [37]. In municipal wastewater (turbidity = 29.4–73.3 NTU, COD = 353–527 mg O_2_/L, color at 400 nm = 0.011–0.041 cm^−1^, total coliform per 100 mL = 4 × 10^8^–2.2 × 10^9^), the use of potassium ferrate resulted in the removal of not only turbidity, COD, and color, but also of bacteria (in comparison with the use of ferric sulfate and aluminum sulfate). It was concluded that the application of potassium ferrate in municipal wastewater treatment could remove 50% more color and 30% more COD and inactivate 3-log_10_ more bacteria in comparison with the same or even smaller doses of ferric sulfate and aluminum sulfate. In these studies, the removal of turbidity, COD, and color and the bacterial inactivation (in log_10_ terms) were 94% (80 and 86% for aluminum sulfate and ferric sulfate, respectively), 32% (6 and 16% for aluminum and ferric sulfate, respectively), 92% (50% for aluminum sulfate and ferric sulfate) and >4 (compared to 1 and 1.05 for aluminum sulfate and ferric sulfate, respectively) [38]. 

Potassium ferrate has also been used to remove carcinogenic nitrosamines [39], sulfonamides [40], and other impurities. Experiments showed that nitrosamines, which are potent carcinogens that are widespread in the environment, could be eliminated from wastewater using potassium ferrate; the complete degradation of nitrosamine was confirmed using photospectroscopy [41]. The oxidation of sulfonamide antibiotics with potassium ferrate showed that the maximum degradation rate (90.01% degradation of 0.06 mol/L sulfonamide solution within 30 min) was observed at pH = 3 with a 14:1 Fe(VI):sulfonamide ratio [40]. This compound is also used as a green material for sustainable soil and groundwater remediation processes in conjunction with other chemicals and materials such as nanoparticles, metal oxides, and zeolites [41]. The studies discussed above indicate that potassium ferrate can be used for the oxidation of various impurities in water and wastewater. Additionally, it has the advantage of being manufactured on an industrial scale and is available commercially. 

The Taguchi method (TM) is a robust statistical design method developed by Genichi Taguchi to improve the quality of manufactured goods. It is also applied in environmental engineering, especially in wastewater treatment, to increase the efficiency of the removal of COD, TOC, and other contaminants. The TM has been used for the optimization of chemical coagulation [42], flux parameters in water containing nitrate, nitrite, phosphate, and sulfite [43], synthetic textile wastewater [37], the electrochemical oxidation of Acid Red 18 [44], and many other processes. The TM can optimize processes by reducing the number of experiments and optimizing the conditions for contaminant removal [44]. An alternative method used to optimize wastewater treatment is the response surface methodology (RSM) based on central composite design (CCD), which requires a larger number of experiments than the TM, but offers more advanced statistical analysis of the results. The RSM has been applied to optimize the coagulation of paper recycling wastewater using *Ocimum basilicum* [45], remove organic compounds from printed circuit board wastewater using the UV-Fenton method [46], optimize the electrocoagulation of instant coffee production wastewater [47], and for many other applications. The novelty of this study is the use of two methods of experiment planning and analysis to optimize the tannery wastewater treatment process using potassium ferrate. 

The goal of this study was to determine the most favorable conditions for the treatment of tannery wastewater using K_2_FeO_4_ (Envifer^®^) and to plan and optimize experiments employing the TM, CCD, and RSM. To compare these methods, the same sets of input parameters were employed for both methods. Planning and optimization of the treatment process using the TM approach made it possible to verify the statistical significance and choose the most favorable combinations and values of the input parameters. The use of RSM allowed more precise statistical analysis of experimental data, including the effect of combining the values of particular input parameters upon the final COD value. The RSM approach was shown to be a more flexible method for planning and optimizing the practical implementation of processes in wastewater purification technology, while the TM approach was found to play an important role during the preliminary stage of process optimization [48,49]. In fact, the pros and cons of the treatment of contaminated tannery wastewater by using potassium ferrate as a relatively new and ecological coagulant were presented with the particular focus on the use of an innovative coagulant, a different type of sewage and advanced statistical methods, respectively. 

## 2. Materials and Methods 

### 2.1. Apparatus and Experiment Conditions

All experiments were performed at a constant temperature (19 ± 1 °C, Inolab^®^ pH/Ion/Cond 750 meter and SenTix^®^ 81 electrodes (WTW, Weilheim in Oberbayern, Germany) in beakers containing tested wastewater (500 ± 2.5 mL). The samples were stirred at 250 rpm using a magnetic mixer (MS11, Wigo, Pruszkow, Poland) during oxidation and coagulation and at 50 rpm during flocculation. The appropriate quantity of K_2_FeO_4_ (Envifer^®^) was added to the measured volume of wastewater. The pH was adjusted to the predetermined value using 20% H_2_SO_4_, and the reaction could proceed for the set time. The amount of K_2_FeO_4_ (Envifer^®^, calculated based on K_2_FeO_4_), pH, and reaction time were set as predetermined when planning the experiments. Na_2_SO_3_ was added (0.5 mol/L) to stop the oxidation reaction. Immediately after completion of the oxidation step, the pH value was adjusted to 8.5 ± 0.1 using 20% NaOH or 20% H_2_SO_4_ (when the final pH was greater than 8.5 ± 0.1) in order to precipitate the Fe^3+^ ions as Fe(OH)_3_. Next, 0.5 mL of 0.05% Furoflock CW277 solution was added, and the stirring speed was reduced to 50 rpm. After 1 min, stirring was stopped to sediment the formed precipitate. A sample of the liquid above the precipitate was collected and passed through a 0.45 µm PTFE (polytetrafluoroethylene) syringe filter (Merck Millipore, Burlington, USA) after 15 min. The filtrate was analyzed as described in the Analytical Procedures section.

### 2.2. Chemicals

Envifer^®^ (Nano Iron, Zidlochovice, Czech Republic) was used as the K_2_FeO_4_ source. The content of K_2_FeO_4_ in Envifer^®^ was determined directly before the procedures described in the Analytical Procedures section. Envifer^®^ was fully characterized (UV-VIS spectrum, energy-dispersive X-ray spectroscopy (EDXS) analysis, scanning electron microscopy (SEM) analysis) previously [37]. To stop the oxidation reaction, 0.5 mol/L Na_2_SO_3_ (Avantor^TM^, Gliwice, Poland) was used. To adjust the wastewater sample pH, 20% solutions of H_2_SO_4_ (Avantor^TM^, Gliwice, Poland) and NaOH (Avantor^TM^, Gliwice, Poland) were applied. A 0.05% solution of Furoflock CW277 (Chemische Fabrik Wocklum Gebr. Hertin GmbH & Co. KG, Balve, Germany) was used as the flocculant. All chemicals were of analytical grade. Throughout the experiments distilled water was used. 

### 2.3. Origin and Physicochemical Parameters of the Raw Tannery Wastewater

Raw wastewater from an industrial tannery located in Poland was used throughout the study. The wastewater originated mainly from leather dyeing processes, and was collected every 60 min over a 24 h period from a raw wastewater reservoir using an autosampler. Equal volumes of each of the wastewater aliquots were mixed to obtain an average daily wastewater sample to be applied in further experiments. Table 1 presents selected physicochemical parameters of the raw wastewater.

### 2.4. Analytical Procedures

The chromite titration method was applied to determine the content of K_2_FeO_4_ in Envifer^®^. This method consists of oxidizing Cr(OH)_4_^−^ ions using FeO_4_^2−^ in extremely alkaline conditions, which results in the formation of Fe(OH)_3_, CrO_4_^2−^, and OH^−^.

(1) $$\%\;of\;K_{2}FeO_{4}=\;\frac{c_{Fe\left(II\right)}\;\times V_{Fe\left(II\right)}\;\times M_{K_{2}FeO_{4}}\;\times 100\%}{3000\;\times \;m_{sample}}$$

where $c_{Fe\left(II\right)}$ and $V_{Fe\left(II\right)}$ are the concentration (0.085 mol/L) and the volume (mL) of the standard Mohr’s salt solution, $M_{K_{2}{FeO}_{4}}$ is 198.04 g/mol, and $m_{sample}$ represents the sample weight (g) [50]. The determination of the K_2_FeO_4_ content in Envifer^®^ was also performed spectrophotometrically (Cary^®^ 50 UV-VIS, Varian Inc., Australia) [51]. Hence, an Envifer^®^ sample was dissolved in distilled water, and the volume was adjusted to 100 mL using a volumetric flask. Next, the sample was filtered (0.45 µm) into a quartz cuvette (light path = 10 mm) and the absorbance values at λ = 505 nm was measured immediately. The K_2_FeO_4_ content (%) in Envifer^®^ was calculated using the following formula:

(2) $$\%\;of\;K_{2}FeO_{4}=\;\frac{A\;\times 0.1\;\times M_{K_{2}FeO_{4}}\;\times 100\%}{1070\;\times \;m_{sample}}$$

where *A* is the absorbance at 505 nm, $M_{K_{2}{FeO}_{4}}$ is 198.04 g/mol, 1070 is the molar absorbance coefficient, M^−1^ cm^−1^, and $m_{sample}$ represents the sample weight (g). The TS, SS, and TDS were determined gravimetrically; however, the TDS was measured after sample filtration [52]. The pH values were specified using an Inolab^®^ pH/Ion/Cond 750 meter and SenTix^®^ 81 electrodes (WTW, Weilheim in Oberbayern, Germany) [53]. A PF-11 spectrophotometer (Macherey-Nagel, Dueren, Germany) was employed to determine the wastewater color at λ = 405 nm [54]. The wastewater COD values were evaluated using a miniature version of the dichromate method and the PF-11 spectrophotometer [55]. TOC was assayed using the ready-made Nanocolor^®^ TOC 60 tube test, while the endpoint was determined using the PF-11 spectrophotometer. TOC assessment was performed in two steps: during the first, inorganic carbon (CO_2_) was eliminated from the samples by adding NaHSO_4_ and stirring the sample (500 rpm, 10 min). In the second step, organic compounds were degraded using Na_2_S_2_O_8_ at 120 °C for 120 min, and the changes in the absorbance of the indicator dye (thymol blue, sodium salt solution) were measured spectrophotometrically at λ = 585 nm. The variations in the absorbance of the dye solution absorbance were caused by the release of gaseous carbon dioxide during the decomposition of the organic compounds in the sample [56].

### 2.5. Procedures for Experiment Optimization Using the Taguchi Method 

Initially, the TM was employed to optimize the preliminary treatment of tannery wastewater. The results were analyzed using Statistica 13 (Tibco Software Inc., Palo Alto, CA, USA) and the decrease in the COD value (in g O_2_/L) was used as the criterion to evaluate the effectiveness of the optimization. Three input parameters were analyzed: K_2_FeO_4_ concentration (g/L), pH value, and the duration of the process (min). Additionally, the effect of experimental run parameter was analyzed. The remaining parameters, namely temperature (19 ± 1 °C), stirring speed (250 rpm during the oxidation step and 50 rpm during the flocculation step), and volume of treated wastewater (500 ± 2.5 mL) were constant in all experiments. Several preliminary experiments were performed to specify the range of pH, time, and K_2_FeO_4_ dose for the experiments based on a literature review of different kinds of wastewater [36,37,38] and our own experiences. Additionally, the redox potential for the oxidation reaction in acidic and neutral media (E° = +2.20 V and E° = +0.72 V, respectively) [36] and the COD value of the raw tannery wastewater were considered. The pH of the purified wastewater was assumed to be about 8.5 based on the quantitative precipitation of iron compounds and optimal conditions for coagulation and flocculation processes using a 0.05% solution of Furoflock CW277; thus, oxidation was expected to appear relatively quickly and the consumption for reagents for pH correction was expected to be low. The preliminary analysis indicated appropriate minimum and maximum values for the K_2_FeO_4_ concentration (0.400 and 1.200 g/L), pH (3 and 9), and the timing of the oxidation process (3 and 9 min). Table 2 shows the design of the four experimental set-ups for the three input parameters, each of which was tested using two values and three runs for a total of 12 experiments, which were performed as described in the Apparatus and Experimental Conditions section. 

The obtained data were analyzed statistically using analysis of variation (ANOVA), the expected S/N (signal to noise) ratio was determined, and the mean values of the criterion function (η) were found using the input parameter values. Finally, experimental verification of the model data was conducted using the appropriate values of the input parameters and number of runs. 

### 2.6. Response Surface Methodology 

Next, RSM was carried out using the software Statistica 13 to optimize the tannery wastewater treatment by analyzing the effect of the three input parameters (concentration of K_2_FeO_4,_ (g/L), pH, and process length (min)) on the COD value (g O_2_/L). The values of the independent parameters ranged from 0.400 to 1.200 g/L for the K_2_FeO_4_ concentration, 3 to 9 for pH, and 3 to 9 min for the length of the oxidation process. The values of the remaining parameters, i.e., temperature, stirring speed, and volume of the treated wastewater sample were constant and identical to those used in the TM studies. Table 3 shows the set-up of the 16 experiments for the RSM. 

The obtained experimental results (the arithmetic mean of three runs was adopted) were analyzed statistically; and the influence of the independent parameters (pH, concentration of K_2_FeO_4_ (g/L), and process duration (min)) on the value of the dependent parameter (COD, g O_2_/L) was shown as a response surface graph. Experimental model verification for the three most favorable parameters for raw wastewater treatment was performed using Statistica software. 

## 3. Results and Discussion

### 3.1. Physicochemical Parameters of the Raw Tannery Wastewater and K_2_FeO_4_ (Envifer^®^)

Preliminary determination of selected physicochemical parameters of the raw tannery wastewater revealed that it had a slightly alkaline pH (pH = 8.6) and high values of dissolved substances TS, SS, and color (19,700 mg/L, 1450 mg/L, and 10,950 mg Pt/L, respectively). Additionally, its COD and TOC values (12,560 mg O_2_/L and 4860 mg/L, respectively), indicated a substantial content of organic compounds in the raw wastewater (see Table 1). Similar characteristics of tannery wastewater have been reported in the literature: pH (7–10.7), TS (10,265–19,775 mg/L), SS (915–5300 mg/L), and COD (2155–11,154 mg O_2_/L) [12]. The earlier EDXS analysis of Envifer^®^ showed that it contained 40.1% pure K_2_FeO_4_. An exact physicochemical analysis of Envifer^®^ revealed that it contained 47.31 ± 1.50% K, 15.00 ± 0.45% Fe, and 37.69 ± 5.20% O, along with impurities such as K_2_O and ferrous compounds other than K_2_FeO_4_ (i.e., K_3_FeO_4_ and KFeO_2_), which resulted from the synthetic method used [37]. In addition, SEM characterization showed an inhomogeneous crystalline structure with some of the features of K_2_FeO_4_ crystals (plump, columnar, and cone-shaped growth).

### 3.2. Taguchi Method 

Initial optimization of the wastewater treatment process was conducted using the TM approach [42,57] (see Table 2). Among the 12 experimental runs, the lowest COD values were obtained in experiments 2, 6, and 10 (2850, 2790, and 2710 mg O_2_/L, respectively) for combination 1-2-2 of the input parameters, i.e., pH = 3, K_2_FeO_4_ = 1.200 g/L, and time = 9 min. The largest COD values were obtained in experiments 4, 8, and 12 (8720, 8750, and 8730 mg O_2_/L, respectively) for combination 2-2-1 of the input parameters, i.e., pH = 9, K_2_FeO_4_ = 1.200 g/L, and time = 3 min. These data revealed that COD reduction was more effective at pH = 3 than at pH = 9, which resulted in a greater dose of K_2_FeO_4_ (1.200 g/L) being used and an extended reaction time (9 min). The preliminary results were analyzed statistically using ANOVA to examine the effect of the independent variables pH, K_2_FeO_4_, and time) on the value of the dependent variable (COD). The results demonstrated that the “repetition” parameter was statistically insignificant (*p* > 0.05) (see Table 4).

The obtained values of COD did not vary significantly in subsequent repetitions (i.e., experiments 1, 5, and 9, Table 3). Additionally, the impacts of the other parameters on the COD value were statistically significant (*p* < 0.05), and, thus, they can be assumed to exert a substantial effect on the value of criterion function η in the following order: pH, time, K_2_FeO_4_ (Table 5). The TM minimizes the process variability in response to interfering factors in the assessment of the effectiveness of analyzed processes, while it maximizes the variability in response to the signal factors (signal to noise ratio). Maximizing the criterion function η = S/N enables joint analysis of both criteria. In the case of wastewater treatment process, a mitigation in COD in agreement with criterion function (3) was significant:

(3) $$\eta \;=\;-10\log_{10}(\frac{1}{n}\sum_{t=1}^{n}y_{i}^{2})$$

where *i* is the number of measurements, η is the S/N ratio, *n* is the number of measurements for a particular process, and *y* is the measured feature. In analyzing the process, the criterion “the smaller the better” was applied [58,59,60,61,62,63]. Table 5 presents the expected S/N values under the most favorable conditions, i.e., for pH = 3, K_2_FeO_4_ = 1.200 g/L, and time = 9 min. 

**Figure 1:**
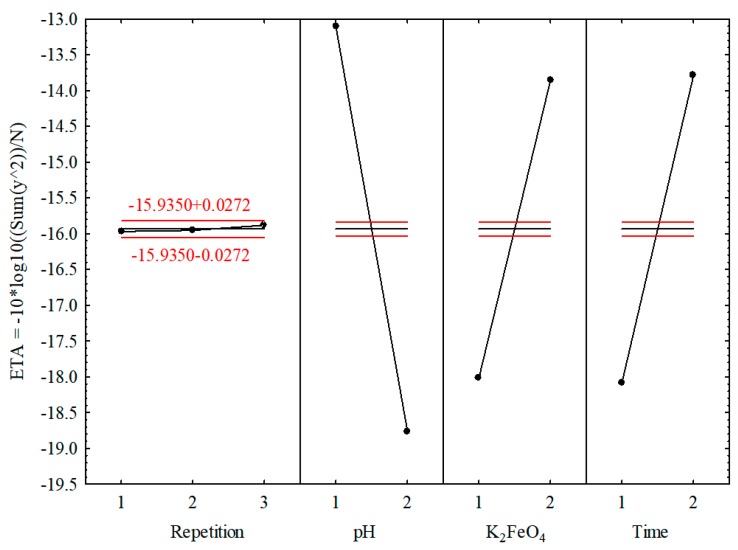
Plot of the ETA (η) values versus the input parameter values for tannery wastewater (pH_1_ = 3.0, pH_2_ = 9.0, K_2_FeO_4_(1) = 0.400 g/L, K_2_FeO_4_(2) = 1.200 g/L, Time(1) = 3.00 min, Time(2) = 9.00 min). The Envifer^®^ dose was calculated based on pure K_2_FeO_4_. The dotted lines give the mean ± 2 × MS Error).

The most valid factor for decreasing the COD value was pH, followed by time and K_2_FeO_4_ (see Table 4 and Table 5). Additionally, only the examined independent parameters were posited to influence the criterion function η = S/N, which was related to the use of orthogonal tables in the TM [64] at the experiment planning stage and the elimination of the possibility of interaction between the independent parameters. Figure 1 presents the relationship between the criterion function η and all the examined parameters. 

The “repetition” parameter did not substantially affect the value of the criterion function, which reached a maximum at pH = 3, K_2_FeO_4_ = 1.200 g/L, and time = 9 (1-2-2). In-depth analysis of the results revealed that the most favorable conditions in terms of decreasing the COD related to the values of the input parameters for which the criterion function η reached a maximum. These values were also consistent with the reference data for the removal of 50 mg/L Reactive Orange 16, in which the pH ranged from 2 to 10 and the concentration of K_2_FeO_4_ was 0.1 g/L. Under these conditions, the highest efficiency of K_2_FeO_4_ in terms of color removal was obtained at an acidic pH (66% at pH = 4); increasing the pH resulted in diminished color removal efficacy (45% at pH = 10) [65]. Reference data for the degradation of toluene using potassium ferrate indicate that the highest toluene removal efficiency was achieved at pH = 6.8 after a period of 20 min. In addition, the oxidation efficiency increased as the molar ratio increased. These studies point out that pH value optimization should take into account a wide range of pH values rather than just pH < 7 [66]. 

The implementation of the TM involves a reference experiment as the last stage, which was performed by executing three runs with the combination of input parameters for which the criterion function η reached maximum values (Figure 1). This combination was identical to that shown in Table 2 for runs 2, 6, and 10, and yielded similar COD values (see Table 6). 

The application of 1.2 g/L K_2_FeO_4_ at pH = 3 caused a depletion in the color (98.4%), the quantity of organic compounds as expressed by the indicators COD and TOC (77.2% and 75.7%, respectively), and the suspended matter (96.9%). The removal efficiencies in a previous study involving wastewater from a carpet factory using conditions optimized to minimize COD (160 mg/L of K_2_FeO_4_, pH = 4) of color and TSS (150 mg/L of K_2_FeO_4_ and pH = 4.5), the removal efficiencies for COD, color, and TSS were 86, 87, and 89%, respectively [36]. The obtained findings were also in line with data for the oxidation of benzophenone-3 during water treatment with potassium ferrate. In this study, it was shown that the second-order rate constant decreased with increasing pH value [67]. These results agreed with the present study, in which better results were achieved at pH = 3 than at pH = 9 for the same time and potassium ferrate concentrations (see Table 3, experiments 1 and 5).

### 3.3. Response Surface Findings

The implementation of CCD and RSM in study planning allowed 16 experiments to be conducted (see Table 3). The obtained COD values (g O_2_/L) related to each experiment are shown in Table 3 (see column 5). The lowest COD values (<3 g O_2_/L) were achieved in experiments 4 and 12 (1.85 and 2.71 g O_2_/L, respectively), while the highest (>10 g O_2_/L) were generated in experiments 5 and 9 (11.8 and 11.15 g O_2_/L, respectively). The lowest COD values were produced in experiments performed with a high dose of K_2_FeO_4_ (1.2 and 1.473 g/L) in an acidic reaction milieu (pH = 3 and 6) and for longer times (6 and 9 min). To find the best conditions in terms of decreasing the COD, the obtained results corresponded to TM results, in which the criterion function η reached maxima at pH = 3, K_2_FeO_4_ = 1.2 g/L, and time = 9 min (Figure 1). Table 7 presents the effects of the normalized values of the independent parameters (pH, K_2_FeO_4_, and time) estimated using ANOVA after the elimination of linear interactions such as pH-K_2_FeO_4_, pH-time, and K_2_FeO_4_-time. The analysis showed that the COD value was mainly affected by the concentration of K_2_FeO_4_ (L) and, additionally, constant value (see Table 7, the first parameter).

**Figure 2:**
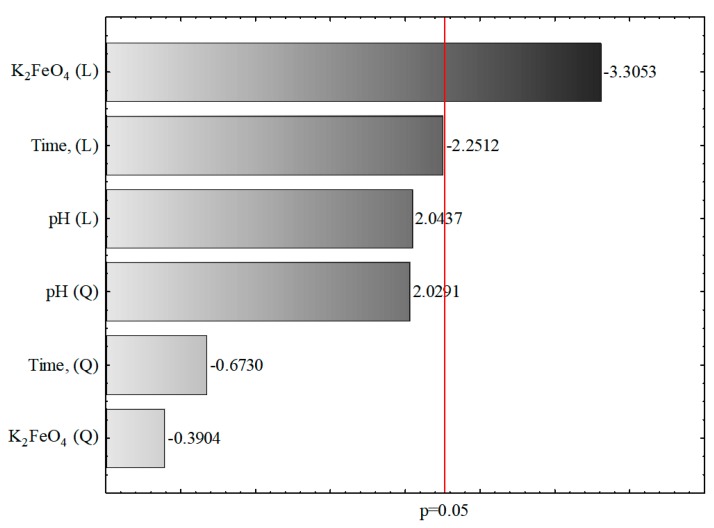
Bar chart of standardized effects (COD, g O_2_/L, 3 value, 1 block, 16 experiments, MS = 3.4812, L—linear effect, Q—quadratic effect, *p*—the absolute value of the standardized effect assessment).

The constant value and K_2_FeO_4_ (L) concentration were determined to be statistically significant (*p* < 0.05), while the pH (L), pH (Q), K_2_FeO_4_ (Q), time (L) and time (Q) were not statistically significant (*p* > 0.05). In addition, the values of the determination coefficient R^2^ and the adjusted determination coefficient R^2^_adj_ (0.77 and 0.59, respectively) illustrated the proportion of the variance in the dependent variable (COD) that was predicted from the independent variables (pH value, concentration of K_2_FeO_4_, time). Previous studies have reported R^2^ and R^2^_adj_ values of 0.96–0.99 and 0.94–0.98, respectively, for wastewater from the carpet industry [36], 0.95 and 0.74 for synthetic textile wastewater [68], and 0.95 and 0.89 for synthetic effluents containing the azo dye Acid Blue 113 [69]. Table 8 shows the results of the adequacy verification of the model coefficients using ANOVA, which corroborated the statistical significance (*p* < 0.05) of the main input parameter K_2_FeO_4_ (L). These results are also presented in a bar chart (Figure 2). 

The estimators of the standardized effects were prioritized according to their absolute value; the vertical line indicates the minimum absolute value for statistical significance. In the tested wastewater samples, K_2_FeO_4_ (L), time (L), and pH (L), and pH (Q) revealed the greatest influence on decreasing the COD value under the experimental conditions, while time (Q) and K_2_FeO_4_ (Q) exerted the smallest effects. 

**Figure 3:**
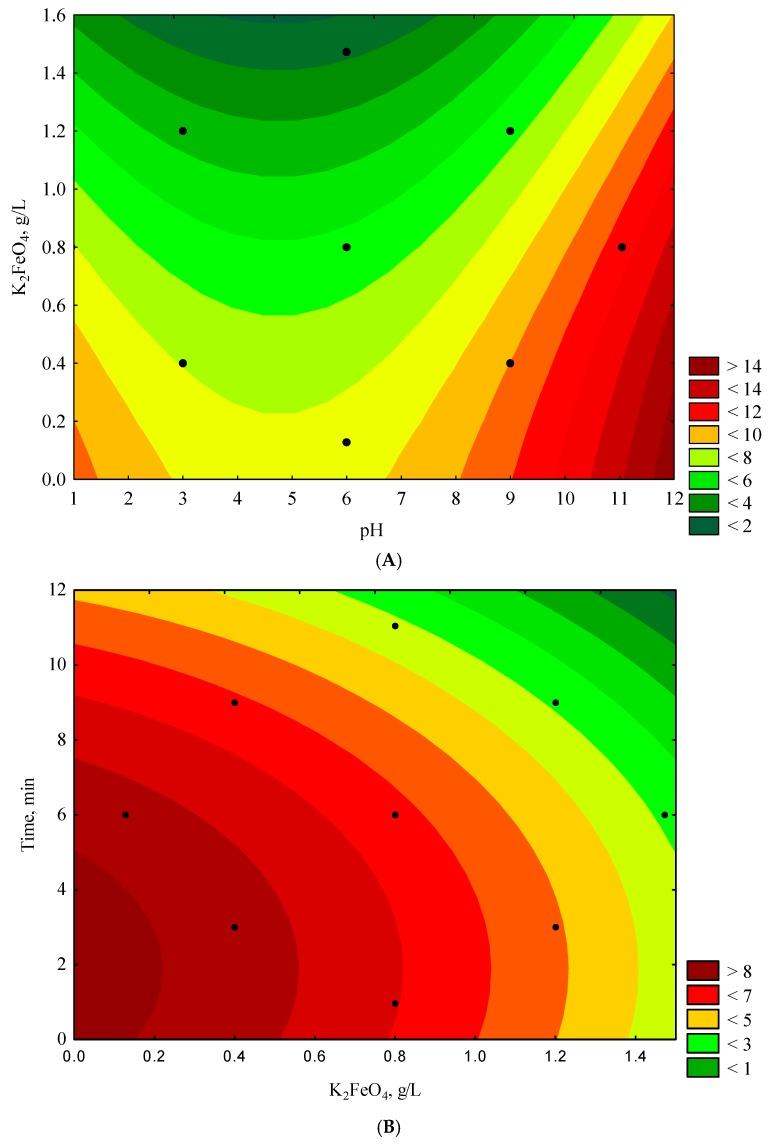

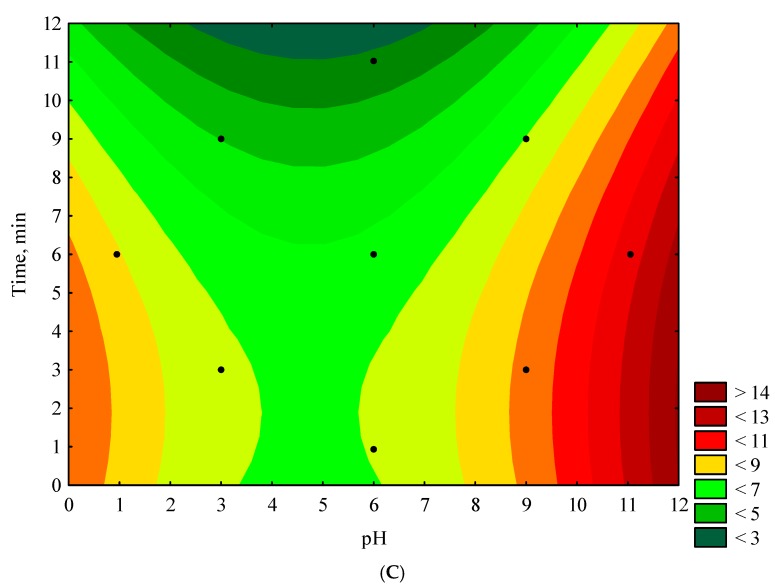
Response surface plots for COD (g O_2_/L) with respect to K_2_FeO_4_ (g/L) and pH (**A**), time (min), and K_2_FeO_4_ (g/L) (**B**) and time (min) and pH (**C**).

The data showed a linear correlation between the experimental and approximated data in the range of tested COD values. Studies using RSM demonstrated (Figure 3A) that the lowest COD value (<2 g O_2_/L) was generated for K_2_FeO_4_ > 1.4 g/L and a pH between 2 and 7.75, with the time parameter set at 6 min.

Accordingly, the greatest efficiency in lowering COD was observed when the input parameters calculated to be most favorable were used. At the same time, for the fixed reaction time of 6 min, the COD values decreased with increasing concentration of K_2_FeO_4_ in the pH range 2–7.75. At a constant pH value of 6 (Figure 3B), the lowest COD values (<1 g O_2_/L) were achieved for time > 9 min and K_2_FeO_4_ > 1.1 g/L. Under these conditions, the effectiveness in terms of lowering COD rose with increasing K_2_FeO_4_ concentration and reaction time. Figure 3C shows the changes in the COD values as a function of time and pH for a constant K_2_FeO_4_ concentration of 0.800 g/L. The lowest COD values (<3 g/L) were produced at a pH between 2.5–7.25 and a reaction time >11 min. Irrespective of the optimum pH values and K_2_FeO_4,_ concentrations, a suitable reaction time was necessary for the oxidation of the organic compounds present in wastewater. In the course of the 16 experiments performed (Table 3), visual discoloration of the examined wastewater was observed within 1–2 min (depending on the experimental set-up) after adding the specified amount of K_2_FeO_4_. However, as demonstrated in our model studies (see Figure 3A–C), a longer reaction time was required to effectively lower the COD (irrespective of the visual discoloration of the wastewater). The pH values, amount of K_2_FeO_4_, and time necessary for the oxidation of the organic compounds present in the wastewater depended on the type and the quantity of the compounds and, often, on those of other compounds that could influence the effectiveness of oxidation processes as well. The best previously reported findings were specified for pH = 6, time = 60 min, and K_2_FeO_4_ = 0.225 g/L (for 25 mg/L methyl orange solution) [70] and pH = 4, time = 20 min, and K_2_FeO_4_ = 0.900 g/L (for synthetic wastewater containing 100 mg/L of the azo dye Reactive Red 2BF) [71]. Results from other studies on the treatment of *m*-cresol wastewater showed that a potassium ferrate dosage of 1.1 g/L, pH value of 5, reaction time of 15 min, and initial m-cresol concentration of 200 mg/L were the optimal conditions, and resulted in a COD removal rate was over 67% [72]. In the present study, for the reference test, the following input parameters were used: pH = 4.5 (Figure 3A,C), K_2_FeO_4_ = 1.400 g/L (Figure 3A,B), and time = 9 min (Figure 3B,C). The dependent variable was calculated (COD = 1.510 g O_2_/L), and the reference experiment was repeated three times (see Table 9).

The color and organic compound content of the wastewater were significantly decreased using K_2_FeO_4_ (↓color 99.3, ↓COD 86.1%, ↓TOC 80.5%). The decreases in these parameters were also greater (see column 3 in Table 6 and Table 9) than those achieved using the TM (↓color 98.4, ↓COD 77.2%, ↓TOC 75.7%). Previously, reported data have also confirmed that the use of K_2_FeO_4_ significantly decreased the level of COD by 86% [36], 32% [38], and 48.5–78.2% in papermaking wastewater and by 85.5% in tannery wastewater [73]. It should be emphasized that the decrease in COD, TOC and color may also result from other chemical compounds in K_2_FeO_4_ (Envifer^®^), commercial product, and that the organic compounds present in wastewater may have been eliminated in part by coagulation and flocculation of the precipitated residue. Under the conditions adopted for the reference test, 3.5 g/L Envifer^®^ was used, taking into account the content of pure K_2_FeO_4_ in the commercial product (40.1%). Potassium ferrate can oxidize many of the organic and inorganic contaminants present in tannery wastewater. These pollutants were determined analytically, i.e., as TOC, COD (as well as inorganic pollutants) and color. The treatment of tannery wastewater using potassium ferrate involves the oxidation of impurities and followed by their coagulation by iron hydroxide. In this case, the transformation of Fe^+6^ to Fe^+3^ takes place via general reactions (1) and (2):

C_x_H_y_O_z_N_k_…* + 8H^+^ + FeO_4_^2−^ +3e^−^ → C_x_H_y_O_z_N_k_…** + Fe^3+^ + 4H_2_O  E° = +2.20V (4)

C_x_H_y_O_z_N_k_…* + 4H_2_O + FeO_4_^2−^ +3e^−^ → C_x_H_y_O_z_N_k_…** + Fe(OH)_3_↓ + 5OH^−^  E° = +0.72 V (5)

where C_x_H_y_O_z_N_k_…* is the general formula of the organic pollutants and C_x_H_y_O_z_N_k_…** is the general formula of the by-products or final oxidation product [74]. The oxidation of some impurities with simple structures to H_2_O and CO_2_ can be ruled out, especially under acidic conditions.

Usually one method is used to optimize industrial and municipal wastewater treatment (TM or CCD/RSM). The combination of two methods is not commonly applied in the practice; however, it allows an initial optimization in the first step (TM) and determine the exact dependencies between all parameters in the second step (CCD/RSM) as was described in this study. From a technological point of view, it avoids problems with manual optimization by using more experiments number. CCD was used for optimization of tannery wastewater treatment recently, but the optimization was performed according to the NBI (network-based inference) algorithm in a mixed arrangement to determine optimal weights. The optimized conditions from CCD modelling and minimization of GSE (Global Standard Error) were identified. This method allows similar dependencies for optimized parameters compared to CCD/RSM [75]. The application of artificial neural networks (ANNs) to model the performance of a common effluent treatment plant treating tannery wastewater was presented recently as well. In this case, the trained model was able to predict the effluent wastewater quality with a correlation coefficient of 0.97 (R^2^ = 0.999, root mean square error <0.2 and the average relative error <18%). ANNs is a method that operates on different assumptions and methodologies compared to TM and CCD/RSM and requires collecting a large database [76]. The selected findings of color, COD, and TOC removal are reported in Table 10.

## 4. Conclusions

The use of K_2_FeO_4_, which is commercially available as Envifer^®^ (40.1% K_2_FeO_4_), was investigated for the treatment of tannery wastewater that was highly contaminated by a wide range of organic compounds originating from leather dyeing processes. Initially, the TM was applied to plan and optimize the treatment process. Subsequently, a CCD/RSM approach was implemented. The same sets of input parameters were employed for both models, namely pH = 3–9, K_2_FeO_4_ = 0.400–1.200 g/L, and time 3–9 min to compare CCD and RSM approaches. Moreover, the range of the specified parameters was expanded (pH = 0.95–11.05, K_2_FeO_4_ = 0.127–1.473 g/L, and time = 0.96–11.05 min) to comply with the CCD methodology. The planning and optimization of the treatment process via the TM approach enabled the statistical significance of the parameters to be verified, and for the most favorable combinations and values of input parameters to be chosen (1-2-2). Undoubtedly, the greatest asset of this method is that only 12 experiments were necessary, compared to 48 total experiments in the RSM approach, as each of the 16 experiments was repeated three times according to the study plan. The implementation of the TM using 1.200 g/L K_2_FeO_4_ at pH 3 for a time of 9 min led to a decrease in the wastewater color (98.4%), COD and TOC (77.2% vs. 75.7%), and suspended matter (96.9%). The employment of CCD and RSM at the planning stage, and the resulting optimized wastewater treatment using 1.400 g/L K_2_FeO_4_ at pH 4.5 for a time of 9 min also reduced the wastewater color by 99.3% and the organic compound content by 86.1% (COD) and 50.5% (TOC). More precise statistical analysis of the experimental data was allowed using RSM, including the effect of combining the values of particular input parameters on the final COD value. The limitation of the RSM approach versus the TM is the greater number of experiments required. While TM allows the determination of the most favorable combinations of input parameters and the elimination of insignificant ones, the RSM approach is based on surface response plots, and allows more precise analysis of the impact of the individual independent factors over a wide range on the dependent variable. In other words, RSM allows the analysis of independent factors (not necessarily those that have the maximum effects in lowering the COD) to take into account the economic conditions and technical or technological aspects of the adopted wastewater treatment technology. Consequently, the RSM approach is a much more flexible method for planning and optimizing the practical implementation of wastewater treatment technologies. On the other hand, the TM approach has the indisputable advantage of requiring less experiments, which minimizes research costs, enables efficient elimination of factors that do not affect wastewater treatment in a substantial way and, last but not the least, simplifies the statistical analysis of the experimental data. Due to these advantages, the TM plays an important role in the preliminary stage of wastewater treatment process optimization.

## Figures and Tables

**Table 1:** Selected physicochemical parameters of the raw tannery wastewater.

Parameter	Unit	Result *
pH	-	8.6 ± 0.1
Total Dissolved Solids	mg/L	18,250 ± 1825
Total Solids	mg/L	19,700 ± 1970
Suspended Solids	mg/L	1450 ± 145
Color	mg Pt/L	10,950 ± 2190
Chemical Oxygen Demand	mg O_2_/L	12,560 ± 1880
Total Organic Carbon	mg/L	4860 ± 729

* parameter value ± the measurement uncertainty for an extension factor k = 2.

**Table 2:** Experimental conditions (factors and levels of the orthogonal array) and results (COD as value±expanded uncertainty) for tannery wastewater (pH_1_ = 3.0, pH_2_ = 9.0, K_2_FeO_4_(1) = 0.400 g/L, K_2_FeO_4_(2) = 1.200 g/L, time(1) = 3.00 min, time(2) = 9.00 min). The dose of Envifer^®^ was calculated based on pure K_2_FeO_4_.

Run	Experimental Conditions							Experimental Results *
	Repetition	pH	K_2_FeO_4_	Time	pH	K_2_FeO_4_ (g/L)	Time (min)	COD (g O_2_/L)
1	1	1	1	1	3.0	0.400	3.00	7.340 ± 1.100
2	1	1	2	2	3.0	1.200	9.00	2.850 ± 0.430
3	1	2	1	2	9.0	0.400	9.00	8.570 ± 1.290
4	1	2	2	1	9.0	1.200	3.00	8.720 ± 1.310
5	2	1	1	1	3.0	0.400	3.00	7.390 ± 1.110
6	2	1	2	2	3.0	1.200	9.00	2.790 ± 0.420
7	2	2	1	2	9.0	0.400	9.00	8.600 ± 1.290
8	2	2	2	1	9.0	1.200	3.00	8.750 ± 1.310
9	3	1	1	1	3.0	0.400	3.00	7.340 ± 1.100
10	3	1	2	2	3.0	1.200	9.00	2.710 ± 0.410
11	3	2	1	2	9.0	0.400	9.00	8.640 ± 1.300
12	3	2	2	1	9.0	1.200	3.00	8.730 ± 1.310

* parameter value ± the measurement uncertainty for an extension factor k = 2.

**Table 3:** Experimental conditions for the RSM and results (COD) for tannery wastewater (K_2_FeO_4_ 0.127–1.473 g/L, pH 0.95–11.05, Time 0.96–11.05 min). The Envifer^®^ dose was calculated based on pure K_2_FeO_4_. (C)—center of plan.

Run	Experimental Conditions			Experimental Results *
	pH	K_2_FeO_4_ (g/L)	Time (min)	COD (g O_2_/L)
1	3.00	0.400	3.00	7.340 ± 1.100
2	3.00	0.400	9.00	5.570 ± 0.840
3	3.00	1.200	3.00	3.350 ± 0.500
4	3.00	1.200	9.00	1.850 ± 0.280
5	9.00	0.400	3.00	11.800 ± 1.770
6	9.00	0.400	9.00	8.570 ± 1.290
7	9.00	1.200	3.00	8.720 ± 1.310
8	9.00	1.200	9.00	6.980 ± 1.050
9	0.95	0.800	6.00	11.150 ± 1.670
10	11.05	0.800	6.00	8.850 ± 1.330
11	6.00	0.127	6.00	8.900 ± 1.340
12	6.00	1.473	6.00	2.710 ± 0.410
13	6.00	0.800	0.96	7.480 ± 1.120
14	6.00	0.800	11.05	3.150 ± 0.470
15 (C)	6.00	0.800	6.00	6.280 ± 0.940
16 (C)	6.00	0.800	6.00	6.270 ± 0.940

* parameter value ± the measurement uncertainty for an extension factor k = 2.

**Table 4:** Analysis of experiment (ANOVA) by using Statistica 13.

Effect/Factor	Analysis of Variance, Mean = −15.9350, Sigma = 4.2932				
	SS	df	MS	F	p
Repetition	0.0180	2	0.0090	0.646	0.5569
pH	95.6632	1	95.6632	6855.424	<0.05
K_2_FeO_4_	51.8397	1	51.8396	3714.937	<0.05
Time	55.1406	1	55.1405	3951.489	<0.05
Error	0.0837	6	0.0136	–	–

Mean—mean of ETA-value, Sigma—population standard deviation, SS—sum of squares, MS—mean square error, F—statistics, df—number of degrees of freedom, p—statistically significant if *p* < 0.05.

**Table 5:** S/N ratio under the optimal conditions.

Effect/Factor	Predicted S/N Ratios under the Optimal Conditions Mean = −15.9350, Sigma = 4.2932		
	Parameter Value	Significance of Effect	Standard Error
Repetition	3	0.0540	0.0591
pH	1 (pH = 3)	2.8235	0.0482
K_2_FeO_4_	2 (1.200 g/L)	2.0785	0.0482
Time	2 (9 min)	2.1436	0.0482
Expected S/N ratio	-	−8.8354	-

Mean—mean of ETA-value, Sigma—population standard deviation.

**Table 6:** Selected physicochemical parameters of treated tannery wastewater after TM application.

Parameter	Unit	Results *	Effect (%) **
pH	-	8.5 ± 0.1	Non-significant
Total Dissolved Solids	mg/L	19,430 ± 1940	↑6.5
Total Solids	mg/L	19,920 ± 1990	↑1.1
Suspended Solids	mg/L	45.0 ± 4.5	↓96.9
Color	mg Pt/L	175 ± 35	↓98.4
Chemical Oxygen Demand	mg O_2_/L	2860 ± 430	↓77.2
Total Organic Content	mg/L	1180 ± 180	↓75.7

* parameter value ± the measurement uncertainty for an extension factor k = 2, ** Effect = $\frac{\left(C1-C2\right)\times 100\%}{C1}$ (for SS, color, COD, and TOC) and ** Effect = $\frac{\left(C2-C1\right)\times 100\%}{C1}$ (for TDS and TS), where c_1_ is the concentration in the raw tannery wastewater, c_2_ is the concentration in the treated wastewater, ↑ represents an increase in the parameter values, and ↓ represents a decrease in the parameter values.

**Table 7:** Analysis of the experiments using CCD with Statistica 13—evaluation of effects.

Parameter	Evaluation of Effects, COD, g O_2_/L, R^2^ = 0.7565, R^2^*_adj_* = 0.5941, 3 Parameter, 1 Block, 16 Experiments, MS = 3.4812								
	Effect	Standard Error	*p*-Value *	−95% Confidence Interval	+95% Confidence Interval	Factor	Standard Error of Factor	Lower Confidence Interval	Upper Confidence Interval
Constant value	6.3052	1.3155	0.0010	3.3295	9.2811	6.3053	1.3155	3.3295	9.2811
pH (L)	2.0637	1.0098	0.0713	−0.2205	4.3480	1.0319	0.5049	−0.1103	2.1740
pH (Q)	2.4877	1.2260	0.0730	−0.2857	5.2612	1.2439	0.6130	−0.1429	2.6306
K_2_FeO_4_ (L)	−3.3375	1.0098	0.0092	−5.6218	−1.0533	−1.6688	0.5049	−2.8109	−0.5267
K_2_FeO_4_ (Q)	−0.4786	1.2260	0.7054	−3.2520	2.2949	−0.2393	0.6130	−1.6260	1.1474
Time (L)	−2.2732	1.0098	0.0509	−4.5574	0.0111	−1.1366	0.5049	−2.2787	0.0055
Time (Q)	−0.8251	1.2260	0.5179	−3.5985	1.9484	−0.4125	0.6130	−1.7993	0.9742

L—linear effect, Q—quadratic effect, * statistically significant if *p* < 0.05.

**Table 8:** Analysis of the CCD experiment using Statistica 13–verification of the adequacy of the model using ANOVA.

Parameter	Evaluation of Effects, COD, g O_2_/L, R^2^ = 0.7565, R^2^*_adj_* = 0.5941, 3 Parameter, 1 Block, 16 Experiments, MS = 3.4812			
	SS	MS	F	P *
pH (L)	14.5408	14.5408	4.1769	0.0713
pH (Q)	14.3335	14.3335	4.1173	0.0730
K_2_FeO_4_ (L)	38.0320	38.0320	10.9249	0.0092
K_2_FeO_4_ (Q)	0.5305	0.5305	0.1524	0.7054
Time (L)	17.6422	17.6422	5.0678	0.0509
Time (Q)	1.5766	1.5766	0.4529	0.5179
Error	31.3312	3.4812	-	-

L—linear effect, Q—quadratic effect, SS—predicted residual error of sum of squares, MS—mean square error, F—statistics, * statistically significant if *p* < 0.05.

**Table 9:** Selected physicochemical parameters of treated tannery wastewater after RSM application.

Parameter	Unit	Result *	Effect (%) **
pH	-	8.5 ± 0.1	Non-significant
Total Dissolved Solids	mg/L	19,590 ± 1960	↑7.3
Total Solids	mg/L	20,560 ± 2060	↑4.4
Suspended Solids	mg/L	30.0 ± 3.0	↓97.9
Color	mg Pt/L	80 ± 16	↓99.3
Chemical Oxygen Demand	mg O_2_/L	1740 ± 260	↓86.1
Total Organic Carbon	mg/L	950 ± 140	↓80.5

* parameter value ± the measurement uncertainty for an extension factor k = 2, ** Effect = $\frac{\left(C1-C2\right)\times 100\%}{C1}$ (for SS, color, COD, and TOC) and ** Effect = $\frac{\left(C2-C1\right)\times 100\%}{C1}$ (for TDS and TS), where *c*_1_-concentration in raw tannery wastewater, *c*_2_-concentration in treated wastewater, ↑-increase in the parameter value, ↓-decrease in the parameter value.

**Table 10:** The removal of color, COD, and TOC from tannery wastewater by using selected methods.

Parameter	Results (This Study)	Results (Other Studies)
COD	↓86.1% (oxidation using K_2_FeO_4_)	↓78.7 ± 1.3% (carbon felt); ↓93.8 ± 1.7% (LTA zolite-modified anode); ↓96.3 ± 2.1% (bentonite-modified anode); bioelectrocatalytic oxidation [77]
		↓80–87% (UF membranes); ↓65% (MF membranes); ↓96% (RO; reverse osmosis) [78]
		↓96.33% (primary treatment process); ↓99.81% (NF; nanofiltration); ↓99.84% (RO; reverse osmosis) [79]
TOC	↓80.5% (oxidation using K_2_FeO_4_)	↓52%; simulated tannery wastewater (MF membranes) [78]; ↓87%; (hydrodynamic cavitation with addition of H_2_O_2_) [80]; ↓50.0%; (coagulation-flocculation/adsorption; CF-ADS) [81]; ↓46.5%; (coagulation-flocculation/ozonation; CF-OZ) [81]
Color	↓99.3% (oxidation using K_2_FeO_4_)	↓61.13%; (coagulation-flocculation/adsorption; CF-ADS) [81] ↓85.34%; (coagulation-flocculation/ozonation; CF-OZ) [81] ↓87%; (electrochemical oxidation; EO) [82]

## References

[1] Leta S., Assefa F., Gumaelius L., Dalhammar G. (2004). Biological nitrogen and organic matter removal from tannery wastewater in pilot plant operations in Ethiopia. Appl. Microbiol. Biotechnol..

[2] Lefebvre N., Vasudevan N., Torrijosa M., Thanasekaran K., Moletta R. (2006). Anaerobic digestion of tannery soak liquor with an aerobic post-treatment. Water Res..

[3] Haydar S., Aziz A. (2009). Characterization and treatability studies of tannery wastewater using chemically enhanced primary treatment (CEPT)-a case study of Saddiq Leather Works. J. Hazard. Mater..

[4] Wang K., Li W., Gong X., Li X., Liu W., He C., Wang Z., Minh Q.N., Chen C.L., Wang J.Y. (2014). Biological pretreatment of tannery wastewater using a full-scale hydrolysis acidification system. Int. Biodeterior. Biodegrad..

[5] Lofrano G., Meric S., Zengin G.E., Orhon D. (2013). Chemical and biological treatment technologies for leather tannery chemicals and wastewaters: A review. Sci. Total Environ..

[6] Lofrano G., Aydin E., Russo F., Guida M., Belgiorno V., Meric S. (2008). Characterization, fluxes and toxicity of leather tanning bath chemicals in a large tanning district area (IT). Water Air Soil Pollut. Focus.

[7] Mannucci A., Munz G., Mori G., Lubello C. (2010). Anaerobic treatment of vegetable tannery wastewaters: A review. Desalination.

[8] De Nicola E., Meriç S., Gallo M., Iaccarino M., Della Rocca C., Lofrano G. (2007). Vegetable and synthetic tannins induce hormesis/toxicity in sea urchin early development and in algal growth. Environ. Pollut..

[9] Munz G., De Angelis D., Gori R., Mori G., Casarci M., Lubello C. (2009). The role of tannins in conventional angogated membrane treatment of tannery wastewater. J. Hazard. Mater..

[10] Tunay O., Kabdasli I., Orhon D., Ates E. (1995). Characterization and pollution profile of leather tanning industry in Turkey. Water Sci. Technol..

[11] Boujelben R., Ellouze M., Sayadi S. (2019). Detoxification assays of Tunisian tannery wastewater under nonsterile conditions using the filamentous fungus Aspergillus niger. BioMed Res. Int..

[12] Durai G., Rajasimmam M. (2011). Biological treatment of tannery wastewater: A review. J. Environ. Sci. Technol..

[13] Kongjao S., Damronglerd S., Hunsom M. (2008). Simultaneous removal of organic and inorganic pollutants in tannery wastewater using electrocoagulation technique. Korean J. Chem. Eng..

[14] Kumar V., Majumdar C., Roy P. (2008). Effects of endocrine disrupting chemicals from leather industry effluents on male reproductive system. J. Steroid Biochem. Mol. Biol..

[15] Iqbal M., Abbas M., Nisar J., Nazir A., Qamar A.Z. (2019). Bioassays based on higher plants as excellent dosimeters for ecotoxicity monitoring: A review. Chem. Int..

[16] Abbas M., Adil M., Ethisham-ul-Haque S., Munir B., Yameen M., Ghaffar A., Shar G.A., Tahir M.A., Iqbal M. (2018). Vibrio fischeri bioluminescence inhibition assay for ecotoxicity assessment: A review. Sci. Total Environ..

[17] Iqbal M. (2016). Vicia faba bioassay for environmental toxicity monitoring: A review. Chemosphere.

[18] Montalvao M.F., de Souza J.M., Guimaraes A.T.B., Menezes I.P.P., Castro A.L.S., Rodriugues A.S.L., Malafaia G. (2017). The genotoxicity and cytotoxicity of tannery wastewater effluent in bullfrog (*Lithobates catesbeianus*). Chemosphere.

[19] Yadav A., Raj A., Purchase D., Ferreira L.F.R., Saratale G.D., Bharagava R.N. (2019). Phytotoxicity, cytotoxicity and genotoxicity evaluation of organic and inorganic pollutants rich tannery wastewater from a Common Effluent Treatment Plant (CETP) in Unnao district, India using *Vigna radiata* and *Allium cepa*. Chemosphere.

[20] Carpenter J., Sharma S., Sharma A.K., Verma S. (2013). Adsorption of dye by using the solid waste from leather industry as an adsorbent. Int. J. Eng. Sci. Invent..

[21] Dixit S., Yadav A., Dwivedi P.D., Das M. (2015). Toxic hazards of leather industry and technologies to combat threat: A review. J. Clean. Prod..

[22] Bhatnagar M.K., Singh R., Gupta S., Bhatnagar P. (2013). Study of tannery effluents and its effects on sediments of river Ganga in special reference to heavy metals at Jajmau, Kanpur, India. J. Environ. Res. Dev..

[23] Money C.A. (2008). Salinity Reduction in Tannery Effluents in India and Australia.

[24] Schilling K., Ulrike B., Helmut K., Zessner M. (2012). Adapting the Austrian Edict on wastewater emissions for tanneries as consequence of foam formation on surface waters. Environ. Sci. Pollut..

[25] Song Z., Williams C.J., Edyvean R.G.J. (2004). Treatment of tannery wastewater by chemical coagulation. Desalination.

[26] Lofrano G., Meric S., Inglese M., Nikolaou A.D., Belgiorno V. (2010). Fenton oxidation treatment of tannery wastewater and tanning agents: Synthetic tannin and nonylphenol ethoxylate based degreasing agents. Desalin. Water Treat..

[27] Iqbal M., Muneer M., Hussain S., Parveen B., Javed M., Rehman H., Waquas M., Abid M.A. (2019). Using combined UV and H_2_O_2_ treatments to reduce tannery wastewater pollution load. Pol. J. Environ. Stud..

[28] Shanmugam B.K., Easwaran S.N., Mohanakrishnan A.S., Kalyanaraman C., Mahadevan S. (2019). Biodegradation of tannery dye effluent using Fenton’s reagent and bacterial consortium: A biocalorimetric investigation. J. Environ. Manag..

[29] Rodriquez-Rodriguez J., Ochando-Pulido J.M., Martinez-Ferez A. (2019). The effect of pH in tannery wastewater by Fenton vs. heterogeneous Fenton process. Chem. Eng. Trans..

[30] Sekaran G., Chitra K., Mariappan M., Raghavan K.V. (1996). Removal of sulphide in anaerobically treated tannery wastewater by wet air oxidation. J. Environ. Sci. Health A.

[31] Krishnomoorhi S., Sivakkumar V., Saravanan K., Prabhu S. (2009). Treatment and reuse of tannery waste water by embedded system. Mod. Appl. Sci..

[32] Mohammed K., Sahu O. (2019). Recovery of chromium from tannery industry waste water by membrane separation technology: Health and engineering aspects. Sci. Afr..

[33] Roopa D., Divya R., Nathiya S. (2019). Management of RO reject water from the tannery industry by solar tunnel dryer. Int. J. Adv. Res..

[34] Tare V., Gupta S., Bose P. (2003). Case studies on biological treatment of tannery effluents in India. J. Air Waste Manag. Assoc..

[35] Audette R.J., Quail J.W., Smith P.J. (1971). Ferrate (VI) ion, a novel oxidizing agent. Tetrahedron Lett..

[36] Moradnia M., Panahifard M., Dindarlo K., Jamali H.A. (2016). Optimizing potassium ferrate for textile wastewater treatment by RSM. Environ. Health Eng. Manag. J..

[37] Thomas M., Barbusinski K., Klis S., Szpyrka E., Chyc M. (2018). Synthetic textile wastewater treatment using potassium ferrate(VI)—Application of Taguchi method for optimisation of Experiment. Fibres Text. East. Eur..

[38] Jiang J.Q., Panagoulopoulos A., Bauer M., Pearce P. (2006). The application of potassium ferrate for sewage treatment. J. Environ. Manag..

[39] Bartzat R., Nagel D. (1991). Removal of nitrosamines from wastewater by potassium ferrate. Arch. Environ. Health Int. J..

[40] Wu K., Wang H., Zhou C.h., Amina Y., Si Y. (2018). Efficient oxidative removal of sulfonamide antibiotics from the wastewater by potassium ferrate. J. Adv. Oxid. Technol..

[41] Rai P.K., Lee J., Kailasa S.K., Kwon E.E., Tsang Y.F., Ok Y.S., Kim K.H. (2018). A critical review of ferrate(VI)-based remediation of soil and groundwater. Environ. Res..

[42] Gokkus O., Yildiz Y.S., Yavuz B. (2012). Optimization of chemical coagulation of real textile wastewater using Taguchi experimental design method. Desalin. Water Treat..

[43] Madaeni S.S., Koocheki S. (2006). Application of Taguchi method in the optimization of wastewater treatment using spiral-wound reverse osmosis element. Chem. Eng. J..

[44] Yousefi Z., Zafarzadeh A., Ghezel A. (2019). Applicaion of Taguchi’s experimental design method for optimization of Acid Red 18 removal by electrochemical oxidation process. Environ. Health Eng. Manag..

[45] Mosaddegh M.R., Shariati F.P., Yazdi S.A.V., Bidhendi G.N. (2018). Application of response surface methodology (RSM) for optimizing coagulation process of paper recycling wastewater using *Ocimum basilicum*. Environ. Technol..

[46] Thomas M., Białecka B., Zdebik D. (2017). Removal of organic compounds from wastewater originating from the production of printed circuit boards by UV-Fenton method. Arch. Environ. Prot..

[47] Bui H.M. (2017). Optimization of electrocagulation of instant coffee production wastewater by using the response surface methodology. Pol. J. Chem..

[48] He Y., Huang G., An C., Huang J., Zhang P., Chen X., Xin X. (2018). Reduction of Escherichia Coli using ceramic disk filter decorated by nano-TiO_2_: A low-cost solution for household water purification. Sci. Total Environ..

[49] Kakoi B., Kaluli J.W., Ndiba P., Thiong’o G. (2017). Optimization of Maerua Decumbent bio-coagulant in paint industry wastewater treatment with response surface methodology. J. Clean. Prod..

[50] Schreyer J.M., Thompson G.W., Ockerman L.T. (1950). Oxidation of chromium(III) with potassium ferrate(VI). Anal. Chem..

[51] Wei Y.L., Wang Y.S., Liu C.H. (2015). Preparation of potassium ferrate from spent steel pickling liquid. Metals.

[52] (1978). PN-C-04541:1978. Water and Wastewater. Determination of Dry Residue, Roasting Residue, Loss after Roasting, Dissolved Substances, Mineral Dissolved Substances and Volatile Dissolved Substances.

[53] (2012). PN-ISO 10523:2012. Water Quality. Determination of pH.

[54] (2011). PN-ISO 7887:2012. Water Quality. Examination and Determination of Colour..

[55] (2005). PN-ISO 15705:2005. Water Quality. Determination of the Chemical Oxygen Demand Index (ST-COD). Small-Scale Sealed-Tube Method.

[56] Nanocolor. http://ftp.mn-net.com/english/Instruction_leaflets/NANOCOLOR/985094en.

[57] Sreeja P.H., Sosamony K.J. (2016). A comparative study of homogeneous and heterogeneous photo-fenton process for textile wastewater. Proc. Technol..

[58] Andrew Liou Y.H., Lin P.P., Lindeke R.R., Chiang H.D. (1993). Tolerance specification of robot kinematic parameters using an experimental design technique the Taguchi method. Robot. Comput. Integr. Manuf..

[59] Thirugnanasambandham K., Sivakumar V., Shine K. (2016). Optimization of reverse osmosis treatment process to reuse the distillery wastewater using Taguchi design. Desalin. Water Treat..

[60] Thirugnanasambandham K., Sivakumar V. (2015). Enzymatic catalysis treatment method of meat industry wastewater using lacasse. J. Environ. Health Sci. Eng..

[61] Thirugnanasambandham K., Sivakumar V., Prakash Maran J. (2015). Performance evaluation and optimization of electrocoagulation process to treat grey wastewater. Desalin. Water Treat..

[62] Thirugnanasambandham K., Sivakumar V. (2015). An eco-friendly approach for copper (II) ion adsorption onto cotton seed cake and its characterization: Simulation and validation. J. Taiwan Inst. Chem. Eng..

[63] Thirugnanasambandham K., Sivakumar V. (2015). Optimization of treatment of grey wastewater using electro-Fenton technique—Modeling and validation. Process Saf. Environ..

[64] Srinu Naik S., Pydi Setty Y. (2011). Optimization for denitrification of wastewater using fluidized bed bioreactor by Taguchi method. Int. J. Biotechnol. Appl..

[65] Sahinkaya S. (2017). Decolorization of reactive orange 16 via ferrate(VI) oxidation assisted by sonication. Turk. J. Chem..

[66] Majid D., Kim I.K. (2018). Degradation of toluene by liquid Ferrate(VI) and solid Ferrate(VI) in aqueous phase. J. Environ. Eng..

[67] Yang B., Ying G.G. (2013). Oxidation of benzophenone-3 during water treatment with ferrate(VI). Water Res..

[68] Thomas M., Barbusiński K., Kalemba K., Piskorz P.J., Kozik V., Bak A. (2017). Optimization of the Fenton oxidation of synthetic textile wastewater using response surface methodology. Fibres Text. East. Eur..

[69] Saravanathamizhan R., Mohan N., Balasubramanian N., Ramamurthi V., Ahmed Basha C. (2007). Evaluation of electro-oxidation of textile effluent using response surface methods. CLEAN Soil Air Water.

[70] Lei B., Zhou C., Cheng T., Du J. (2013). Synthesis of Potassium Ferrate by chemical dry oxidation and its properties in degradation of methyl orange. Asian J. Chem..

[71] Dong X.L., Wang L., Zhang X.X., Bai L., Zhang X.F., Ma H.C., Ma C., Xue M. (2012). Oxidative degradation of azo dye reactive red 2BF by Potassium Ferrate. Adv. Mater. Res..

[72] Hu M.Z., Shi Z.H., Zhao H.Y. (2013). Study on treatment of m-cresol wastewater using potassium ferrate(VI). Adv. Mater. Res..

[73] Mia Z., Wang F., Deng D., Wang L., Yang J. (2012). Removal effect of Potassium Ferrate to COD in different wastewater. Adv. Mater. Res..

[74] Jiang J.Q., Stanford C., Petri M. (2018). Practical application off Ferrate(VI) for water and wastewater treatment—Site study’s approach. Water Energy Nexus.

[75] Pinto B.M., Samanamud G.R.L., Baston E.P., Franca A.B., Naves L.L.R., Lourdes C.C.A., Naves F.L. (2019). Multivariate and multiobjective optimization of tannery industry effluent treatment using Musa sp flower extract in the coagulation and flocculation process. J. Clean. Prod..

[76] Priya K., Abbasi T., Murugaiyn V. (2018). Modeling the performance of a tannery common effluent treatment plant using artificial neural networks. Desalin. Water Treat..

[77] Elabed A., El Khalfaouy R., Ibnsouda S., Basseguy R., Elabed S., Erable B. (2019). Low-cost electrode modification to upgrade the bioelectrocatalytic oxidation of tannery wastewater using acclimated activated sludge. Appl. Sci..

[78] Zouboulis A.I., Peleka E.N., Ntolia A. (2019). Treatment of tannery wastewater with vibratory shear-enhanced processing membrane filtration. Separations.

[79] Stoller M., Sacco O., Sannino D., Chianese A. (2013). Successful integration of membrane technologies in a conventional purification process of tannery wastewater streams. Membranes.

[80] Korpe S., Bethi B., Sonawane S.H., Jayakumar K.V. (2019). Tannery wastewater treatment by cavitation combined with advanced oxidation process (AOP). Ultrason. Sonochem..

[81] Mella B., de Carvalho Barcellos B.S., da Silva Costa D.E., Gutterres M. (2018). Treatment of leather dyeing wastewater with associated process of coagulation-flocculation/adsorption/ozonation. Ozone Sci. Eng..

[82] Sathishkumar K., Narenkumar J., Madhavan J., Murugan K., Rajasekar A. (2017). Electrochemical decolorization and biodegradation of tannery effluent for reduction of chemical oxygen demand and hexavalent chromium. J. Water Process Eng..

